# *Clostridium difficile* Infections amongst Patients with Haematological Malignancies: A Data Linkage Study

**DOI:** 10.1371/journal.pone.0157839

**Published:** 2016-06-17

**Authors:** Linda A. Selvey, Claudia Slimings, David J. L. Joske, Thomas V. Riley

**Affiliations:** 1 School of Public Health, Curtin University, GPO Box U1987, Perth, WA 6845, Australia; 2 School of Pathology and Laboratory Medicine, The University of Western Australia, Perth, WA, Australia; 3 Department of Haematology, Sir Charles Gairdner Hospital, Nedlands, WA Australia; 4 School of Medicine, University of Western Australia, Perth, WA, Australia; 5 Department of Microbiology, PathWest Laboratory Medicine, Nedlands, WA, Australia; Cleveland Clinic, UNITED STATES

## Abstract

**Objectives:**

Identify risk factors for *Clostridium difficile* infection (CDI) and assess CDI outcomes among Australian patients with a haematological malignancy.

**Methods:**

A retrospective cohort study involving all patients admitted to hospitals in Western Australia with a haematological malignancy from July 2011 to June 2012. Hospital admission data were linked with all hospital investigated CDI case data. Potential risk factors were assessed by logistic regression. The risk of death within 60 and 90 days of CDI was assessed by Cox Proportional Hazards regression.

**Results:**

There were 2085 patients of whom 65 had at least one CDI. Twenty percent of CDI cases were either community-acquired, indeterminate source or had only single-day admissions in the 28 days prior to CDI. Using logistic regression, having acute lymphocytic leukaemia, neutropenia and having had bacterial pneumonia or another bacterial infection were associated with CDI. CDI was associated with an increased risk of death within 60 and 90 days post CDI, but only two deaths had CDI recorded as an antecedent factor. Ribotyping information was available for 33 of the 65 CDIs. There were 19 different ribotypes identified.

**Conclusions:**

Neutropenia was strongly associated with CDI. While having CDI is a risk factor for death, in many cases it may not be a direct contributor to death but may reflect patients having higher morbidity. A wide variety of *C*. *difficile* ribotypes were found and community-acquired infection may be under-estimated in these patients.

## Introduction

*Clostridium difficile* infection (CDI) is a well-recognised nosocomial infection, particularly amongst patients treated with antibiotics. Since 2003, the rate of healthcare-associated CDI (HA-CDI) has escalated in North America and Europe with the emergence of a new virulent strain (PCR ribotype 027/North American pulse-field type 1) [1,2]. Notwithstanding several known introductions to Australia [3,4], this strain has not become established [5]. Despite this, all Australian states have seen a significant increase in the rates of CDI since mid-2011[5]. Increasingly CDI is also recognised as a community acquired infection [5].

The incidence of CDI amongst patients with haematological malignancies is much higher than amongst hospitalised patients with other conditions [6]. Certain malignancies such as acute myeloid leukaemia (AML) [7], procedures such as stem cell transplants [8–11], prolonged neutropenia [7,12], and treatment with particular antibiotics [7,12] have been previously documented as being associated with CDI in this group.

In Australia, there is a lack of information on the incidence of CDI in patients with haematological malignancies, risk factors for CDI and the risk of mortality associated with CDI in this patient group. This is important given the absence of the epidemic *C*. *difficile* PCR ribotype 027 strain in this country. In this study, we reviewed the clinical records of hospitalised patients with haematological malignancy with the following aims: to estimate the incidence of CDI, to identify risk factors for CDI; and to examine whether CDI increases the risk of mortality in patients with haematological malignancies.

## Methods

### Data source

This study was a retrospective cohort study involving all patients admitted at least once to any hospital in WA for treatment or management of a haematological malignancy in the period 1 July 2011 to 30 June 2012. This included patients who were an inpatient on the 1 July 2011 but had been admitted prior to that date. Hospital admission data were obtained from the WA Hospital Morbidity Data System (HMDS) and linked with routinely collected surveillance records of all hospital investigated CDI cases from the Healthcare Associated Infection Surveillance WA (HISWA) program (Healthcare Associated Infection Unit, WA Department of Health) and statutory death notifications. HISWA surveillance data are provided by all public metropolitan, regional and integrated district hospitals (n = 32) and 15 of 17 (88%) private hospitals providing acute care [13]. Data provision is mandatory for all public hospitals and private hospitals that are funded to provide care to public patients [13]. Data collection within hospitals are reviewed for consistency by the Healthcare Associated Infection Unit, and the data are validated as described [13,14]. Statutory death notifications were obtained from the Registry of Births, Deaths and Marriages. Details of the cause of death for each death are provided on death certificates by attending medical practitioners except where the death is investigated by the Coroner [15]. As a statutory death notification system, it is likely that the data are complete, but the quality of the reasons for death information will depend on individual attending medical practitioners. The WA Data Linkage Unit undertook the data linkage as previously described [15].

The following records for each patient were extracted: HISWA records of hospital identified CDI diagnosed from July 2011 to June 2012 (specimen date, source of exposure and *C*. *difficile* ribotype); HMDS records for all hospital discharges from CDI reporting hospitals over the study period (age, gender, hospital category, length of stay, month and year of hospital admission and discharge, diagnosis (International Standard Classification of Diseases and Related Health Problems, 10^th^ Revision, Australian Modification (ICD-10-AM)) and procedure (Australian Classification of Health Interventions (ACHI) 7^th^ edition) codes, and days in ICU); and death notifications to 31 December 2012 for any patient (date of death, whether died in hospital and cause of death (free text)) to capture 6 months’ follow up from the date of CDI infection. From October 2011, viable isolates of *C*. *difficile* were ribotyped using PCR typing [16]. No information on drugs and therapeutic agents was included in this dataset.

### Inclusion criteria

We included all patients who had been admitted to one of the participating hospitals at least once during the study period and who had a haematological malignancy documented as a primary diagnosis for at least one admission. We also included all patients who had been admitted for anaemia, thrombocytopenia, neutropenia or sepsis or received anti-neoplastic chemotherapy or had a bone marrow biopsy and had a haematological malignancy documented as an additional (not primary) diagnosis (1 to 4 only) for at least one admission. All hospital admissions for patients meeting the inclusion criteria were included in the analysis. Haematological malignancies were defined as any conditions with ICD-10-AM codes from C81 (Hodgkins lymphoma) to C96 (Langerhans-cell histiocytosis) inclusive (defined in ICD-10-AM as malignant neoplasms of lymphoid, haematopoietic and related tissue). Unless otherwise stated, patients with malignancies in remission and not in remission were included in the analysis.

### CDI definition

Patients with CDI were defined as recommended by the Healthcare Associated Infection Technical Working Group of the Australian Commission on Safety and Quality in Health Care [17] as having diarrhoea, i.e. an unformed stool taking the shape of the container, and meeting the following criteria: either a faecal sample positive for *C*. *difficile* toxin A and/or B by enzyme immunoassay or tissue culture neutralisation assay; or having toxin-producing *C*. *difficile* detected in a stool sample by PCR or culture. Exclusion criteria were: CDIs occurring within 8 weeks of a previous positive sample, and CDIs in children under 2 years [17]. Only hospital-identified CDI cases were included. These were CDIs diagnosed in a patient attending any part of a hospital (including those attending an emergency department or hospital outpatient departments) [17].

CDI cases were classified according to their likely source of exposure as defined by McDonald and colleagues [18]. CDI cases were classified as health care associated if their symptom onset was 48 hours or more following a hospital admission or their onset occurred outside of a hospital admission or within 48 hours of admission and they had a previous hospital admission in the past 28 days. CDI cases were considered to be indeterminate if their onset occurred outside of a hospital admission or within 48 hours of admission and they had a previous hospital admission between 28 days and 12 weeks, and community acquired if they had no hospital admission within 12 weeks of onset.

### Main Outcomes

#### Mortality

We defined mortality as death within 60 or 90 days of CDI or a proxy date for non-CDI cases. For the non-CDI cases we assigned a proxy date as previously described [19]. To assign a proxy date, CDI cases were matched with all non-CDI cases that had the same malignancy and month of first admission and these non-CDI cases were given a proxy date that was the date of the *C*. *difficile* positive faecal specimen for the corresponding CDI case. Where there was more than one CDI case with the same malignancy and the same month of first admission, all non-CDI cases that matched these CDI cases were randomly assigned proxy dates corresponding to each of the CDI cases using random number generation in SPSS v22 (IBM, New York, NY, USA).

#### Hospital admission

We reviewed the ICD-10-AM primary and additional diagnosis codes for CDI cases for their admission in which CDI was diagnosed and immediate subsequent admissions to determine whether CDI was documented as a reason for admission.

Intra-hospital transfers were not counted as new admissions and the total duration of hospital stay where an intra-hospital transfer occurred was calculated as the duration from the beginning to the end of admission in that hospital. Patient length of stay was defined according to the METEOR definition, which defines same-day patients as having a single day length of stay [20].

### Risk factors

#### Comorbidities

Patients were defined as having comorbidities according to Charlson and colleagues [21], and classified according to ICD-10-AM codes in any of the primary and additional diagnosis codes for any admission using the Charlson’s index macro in STATA v12 (StataCorp LP, College Station, USA) [22].

#### Risk factors for acquiring CDI

We assessed the association of the following potential risk factors with CDI: age, type of malignancy, neutropenia, duration and numbers of hospital admissions (prior to the date of CDI-positive faecal sample), procedures, ICU admissions, and admissions to hospital from long term care facilities. Neutropenia was defined from ICD-10-AM codes (D70) when documented in any diagnosis or additional diagnosis codes. These potential risk factors were selected after a review of the literature [7,8,11,12,23–25]. We also assessed the association of bacterial infections with CDI defined as bacterial sepsis (ICD-10-AM codes A40, A41, A 32.7, A37.7, R65.2), bacterial pneumonia (ICD-10-AM codes J16 to J18), and other bacterial infections (ICD-10-AM codes A40, A41, A48, B95, B96, B99, G00-G03, G06, G07, L00-L08, N30, N39, K65) and assessed the association of all Charlson’s comorbidities with CDI.

#### Risk factors for death within 60 and 90 days of admission

We assessed the following risk factors for their association with death: age, sex, ICU admission, previous admission to a long term care facility, malignancy type, comorbidities, CDI, bacterial sepsis (ICD-10-AM codes A40, A41, A 32.7, A37.7, R65.2), bacteria pneumonia (ICD-10-AM codes J16 to J18), other severe infections (ICD-10-AM codes K65, J09, J10, J12) and neutropenia (ICD-10-AM code D70). The risk factors of malignancy type and comorbidities were also obtained from ICD-10-AM codes.

### Data analysis

Data were analysed using SPSS v22 (IBM, New York, NY, USA) and STATA14.1 (Statacorp LP, College Station, Texas, USA). The incidence of CDI (CDI cases/10,000 BD) was calculated by dividing the total number of CDIs regardless of source of infection by the product of the number of all CDI cases in the cohort and their total duration of hospital stay during the study period and multiplying by 10,000. Chi squared tests or Fisher’s exact tests were used to test for statistical significance in univariate analysis of categorical variables and the Wilcoxon-Mann-Whitney test was used to test the differences between the medians of continuous variables.

#### Logistic regression

To identify risk factors for CDI, two logistic regression models were developed. For the first model, in order to allow for at least 3 months of hospitalisation data prior to CDI, only patients who had at least one hospital admission in October 2011 or later were included. For this analysis, CDI cases were defined as patients with CDI onset from October 2011 onwards. If the only CDI event occurred prior to October 2011 they were not defined as having CDI in the model, but the CDIs were counted in a variable indicating previous CDI. The other model included all patients in the cohort. For this model, any time-related factors such as length of stay or total admissions prior to infection were excluded because the previous admission history in the last 3 months was not available for all patients.

Logistic regression was performed using a forced entry procedure and backwards elimination. Variables were included in the initial model if there was an association with CDI documented in the literature or if univariate analysis of the association between the variable and CDI found a p value of <0.1. Variables were eliminated from the model one by one if the p value for the predictor was greater than 0.1. Interaction terms were included in the initial model and significant interaction terms were retained in the final model. The most parsimonious model was used and model fit was assessed using Nagelkerke R^2^.

#### Cox regression

Cox Proportional Hazards Regression analysis was performed using the duration from CDI to death as the time-dependent variable, and death within 60 and 90 days of CDI as the status of interest. The time to death was calculated as either the censure date (60 or 90 days post-CDI or proxy date) or date of death minus the most recent positive faecal sample date or proxy date. The proxy date was also used to calculate other time-related variables such as duration of hospital stay and numbers of hospital admissions. To adjust for potential confounders, variables were included in the initial model if univariate analysis between the variable and death found a p value <0.1. Variables were eliminated from the model one by one if the p value for the predictor was greater than 0.1. Age was dichotomised to 65 years and over and under 65 and all other variables in the models were binary. The proportional hazards assumption was tested for all variables in the final model visually by plotting log-log Kaplan-Meier survival estimates, log-log Cox adjusted survival estimates and Kaplan-Meier survival estimates and Cox adjusted survival estimates plotted on the same graph. The assumption was also tested using the STATA proportional hazards assumption test, which tests for a correlation between scaled Schoenfeld residuals and ranked failure time. Where the proportional hazards assumption was violated, the regression was stratified by the variable violating the assumption.

### Ethics

The project was approved by the WA Health Ethics Committee (number 2013/35) and by the Curtin University Human Research Ethics Committee (HR 186/2013). Given the impracticality of obtaining consent for inclusion in this study and the limited risk associated with inclusion of participants, a waiver of consent was granted by both Human Research Ethics Committees.

## Results

There were 2085 patients with a haematological malignancy who had been admitted to one of the participating hospitals at least once during the study period, and who met the inclusion criteria. There were an additional 159 patients with a haematological malignancy who did not meet the inclusion criteria and were excluded from further analysis. Of the 159 excluded patients, 50 (31.5%) had a form of chronic lymphoid leukaemia (compared to 7.9% of the included patients) and four developed CDI during the study period. The median age of excluded patients (75) was higher than that of included patients (65), p<0.001.

Of the 2085 included patients, 785 (37.6%) had their first recorded admission in July 2011 (at the beginning of the study period) or before. Ninety seven (4.7%) patients were aged 15 years or less and 1060 (50.8%) patients were aged 65 or above. Sixty five patients (3.1%) had at least one CDI in the study period; one of the 65 had two recurrences and another two each had one recurrence of infection. The overall incidence of CDI was 15.0/10,000 BD.

CDI cases were younger than non-CDI cases and were more likely than patients with no CDI to have been admitted to a tertiary hospital (Table 1). Patients were more likely to have CDI during the study period if they had an autologous stem cell transplant or chemotherapy during that period and 80% of patients with CDI had neutropenia, compared to 22% of patients without CDI (Table 1). There was no difference in the number of admissions prior to CDI infection between patients with and without CDI.

**Table 1:** Characteristics of the study sample by CDI status–potential predictors.

	CDI^1^ (n = 65)	No CDI^2^ (n = 2020)	p value^3^
Female, n (%)	21 (32.2)	865 (42.8)	.098
Median age (interquartile range)	61.0 (49.5, 70.0)	65.0 (53.0, 75.0)	.010
Number of admissions by hospital type (excluding transfers)^4^			
All	1004	17477	
Tertiary, n (%)	801 (79.8)	10580 (60.5)	
Public metropolitan, n (%)	7 (0.7)	429 (2.5)	
Private metropolitan, n (%)	133 (13.2)	4962 (28.4)	
Rural, n (%)	63 (6.3)	1506 (8.6)	<0.001
Median number of admissions^5^ prior to first CDI^6^ (interquartile range)	5.0 (2.0, 11.5)	6.0 (3.0, 11.0)	.901
Median inpatient days prior to first CDI (interquartile range)	29.0 (12.0, 45.5)	13.0 (5.0, 31.0)	<0.001
Any admission from a long term care facility, n (%)	1 (1.5)	32 (1.6)	1.00
Any ICU^7^ admissions prior to CDI^8^, n (%)	3 (4.6)	108 (5.3)	1.00
Diabetes, n (%)	5 (7.7)	121 (6.0)	.591
Neutropenia, n (%)	52 (80.0)	446 (22.1)	<0.001
**Procedures**			
Allogeneic bone marrow transplant, n (%)	3 (4.6)	32 (1.6)	.093
Autologous stem cell transplant, n (%)	7 (10.8)	55 (2.7)	.003
Chemotherapy, n (%)	58 (89.2)	1423 (70.5)	.001
Radiation, n (%)	1 (1.5)	29 (1.4)	.616

^1^ Patients who had *C*. *difficile* infection at least once in the study period

^2^ Patients who had no episodes of *C*. *difficile* infection in the study period

^3^ Percentages were based on number of admissions in each group

^4^ Outcome of the test for significance of difference between patients with and without *C*. *difficile* infection using a Chi squared test or Fisher’s exact test for categorical variables and Wilcoxon-Mann-Whitney for difference in medians.

^5^ Median number of admissions since study commencement

^6^ CDI—*C*. *difficile* infection

^7^ ICU—Intensive care unit

^8^ ICU admissions since study commencement

The median number of admissions for patients with CDI over the study period (12.0, 95% CI 5.5, 23.5) was higher than patients without CDI (6.0, 95% CI 3.0, 11.0), p<0.001. The median total inpatient days was also higher for patients with CDI (58.0, 95% CI 31.5, 97.5) than those without CDI (13.0, 95% CI 5.0, 31.0), p<0.001. A higher proportion of patients with CDI were admitted to ICU over the study period (16.9%) compared to those patients without CDI (5.3%), p = 0.001.

The median age of patients with either AML or ALL (56) was less than patients with other malignancies (66), p<0.001. Sixty one percent of patients with AML, 50% of patients with acute lymphocytic leukaemia (ALL), and 37% of patients with diffuse large B cell lymphoma had neutropenia during at least one hospital admission, which was higher than patients with other malignancies (p<0.001). Patients who had chemotherapy on at least one admission, autologous stem cell transplants or allogeneic bone marrow transplants were also more likely to have neutropenia than other patients, as were patients with liver disease (mild and moderate/severe), congestive heart failure and diabetes (uncomplicated and complicated) (data not shown).

The most common malignancy was non-Hodgkin’s lymphoma (45.9%) (of which 32.4% were patients of diffuse large B cell non-Hodgkin’s lymphoma), followed by multiple myeloma (21.8%). Fourteen percent of patients with ALL not in remission and 7% of patients with AML not in remission developed CDI during the study period, (Table 2).

**Table 2:** Proportion of patients with different haematological malignancies and co-morbidities by *C*. *difficile* infection status.

		CDI^1^	p value^2^
		n (%)	
	Total number of patients	65 (3.1)	
**Type of malignancy**			
Acute myeloid leukaemia no remission (C92)^3^	154	11 (7.1)	.003
Acute myeloid leukaemia in remission^3^	16	1 (6.3)	.469
Acute lymphoblastic leukaemia no remission (C91.0)^3^	66	9 (13.6)	<0.001
Acute lymphoblastic leukaemia in remission^3^	43	3 (7.0)	.141
*Non-Hodgkin’s lymphoma*			
Non-Hodgkin’s lymphoma—follicular (C82)^3^	225	4 (1.8)	.308
Non-Hodgkin’s lymphoma—remaining (C83, C84, C85, C86, C88)^3^	762	21 (2.8)	.515
Hodgkin’s lymphoma (C81)^3^	105	1 (1.0)	.256
Multiple myeloma no remission (C90)^3^	463	10 (2.2)	.179
Multiple myeloma in remission^4^	6	0	.660
Other leukaemia (C92 to C96)^3^	109	3 (2.8)	1.00
**Comorbidities**			
Peptic ulcer	21	3 (14.3)	0.026
Renal disease	121	8 (6.6)	0.023
**Other Infections**			
Any bacterial infection prior to CDI^5^	670	38 (5.7)	<0.001
Sepsis at any time	256	31 (12.1)	<0.001
Bacterial pneumonia at any time	232	16 (6.9)	<0.001
Bacterial infection other than pneumonia at any time	569	46 (8.1)	<0.001
Other severe infection at any time	33	4 (12.1)	0.018

^1^ Patients who had *C*. *difficile* infection at least once in the study period

^2^ Outcome of the test for significance of difference between patients with and without *C*. *difficile* infection using a Chi squared test or Fisher’s exact test for categorical variables

^3^ ICD-10-AM codes

^4^ Patients were only classified as being in remission if they were in remission for the full duration of the study

^5^ CDI–*C*. *difficile* infection

The proportion of patients with different types of malignancies and their CDI status is shown in Table 2. Of the 2085 patients, 63 had been admitted in the study period with more than one haematological malignancy recorded (two of these had three malignancies in the different categories). The most common combinations of malignancy were follicular lymphoma and another non-Hodgkin’s lymphoma (29 patients) and a lymphoma and chronic lymphocytic leukaemia (20 patients).

Of the Charlson’s comorbidities, patients with peptic ulcers and renal disease were more likely to develop CDI than those who did not have these conditions (Table 2). No other Charlson’s comorbidities were significantly associated with CDI (S1 Table). Patients with CDI were more likely to have had another bacterial infection (Table 2).

### Source of acquisition of CDI

Of the 65 CDI cases, two had community-acquired CDI and one had CDI of indeterminate source. The remaining 62 CDI cases were apparently hospital-acquired. Of these 62 cases, there were ten (16%), who within 28 days prior to having a *C*. *difficile* positive faecal sample had only single-day hospital admissions (for example for chemotherapy). Two additional cases only had single-day admissions recorded but their positive faecal sample was taken within 28 days of starting the study and therefore their full admission history within the 28 day period prior to CDI onset was not available.

### Ribotyping

Ribotyping information was available for 33 of the 65 CDIs. There were 19 different RTs identified. The most common RT was 014/020 group (7 cases) followed by 002 (5 cases). There were three cases of 007 and two each of 244 and 070. All other RTs infected one case only (S2 Table).

### CDI outcomes

1. Death within 60 days of CDI

A total of 1137 patients either had CDI or a proxy date assigned. The remaining patients did not have CDI and their malignancy type and month of first admission did not coincide with that of a CDI case and therefore were not assigned a proxy date. 36 of the 1137 patients died within 60 days of the date of most recent CDI or their proxy date. Patients that had CDI (21.5% vs 2.1% p<0.001), were older (median age 73.5 vs 64, p = 0.003), had neutropenia during at least one admission (6.5% vs 1.9%, p<0.001), had AML not in remission (10.8% vs 2.8%, p = 0.003), or were admitted to ICU at any time (11.3% vs 2.9%, p = 0.005) were more likely to die within 60 days of CDI or proxy date. Patients with sepsis (11.5% vs 2.5%, p<0.001), bacterial pneumonia (13.2% vs 2.6%, p<0.001) and any other severe infection (11.5% vs 2.5%, p<0.001) after CDI or proxy date were also more likely to die within 60 days of CDI or proxy date. Patients with the following comorbidities: cerebrovascular disease; dementia; peptic ulcer disease; renal disease; and moderate/severe liver disease were also more likely to die within 60 days of CDI or proxy date (data not shown). There were no patients who had graft vs host disease recorded.

2. Death within 90 days of CDI

A total of 57 of the 1137 patients included in the analysis died within 90 days of CDI or the proxy date. Patients that had CDI (30.6% vs 3.6% p<0.001), were older (median age 74 vs 64, p = 0.002), had neutropenia during at least one admission (9.5% vs 3.5%, p<0.001), had AML (15.4% vs 4.6%, p = 0.001), were admitted to ICU at any time (17.0% vs 4.6%, p = 0.001), or were admitted from a long-term care facility (21.4% vs 5.1%, p = 0.029) were more likely to die within 90 days of CDI or proxy date. Patients with sepsis (19.5% vs 4.0%, p<0.001) or bacterial pneumonia (17.6% vs 4.4%, p<0.001) after CDI or proxy date were also more likely to die within 90 days of CDI or proxy date. Patients with the following comorbidities: cerebrovascular disease; congestive heart failure; dementia; peptic ulcer disease; renal disease; and moderate/severe liver disease were also more likely to die within 90 days of CDI or proxy date (data not shown).

3. Hospital admission

Of the 65 patients with CDI, 40 (61.5%) had CDI recorded as a reason for the hospital admission (in any diagnosis field) during which the *C*. *difficile* positive specimen was taken or the admission following this one. The remainder did not have CDI recorded, suggesting that CDI did not contribute to the reason for admission and subsequent clinical course.

### Multivariate analysis–CDI as the outcome

Model 1 –Patients who had at least one hospital admission in October 2011 or later

There were 1881 patients included in this analysis, of whom 50 developed CDI in October 2011 or later. In the model, having ALL not in remission (Adj OR 3.2), neutropenia (Adj OR 15.6), renal disease (Adj OR 2.8) and peptic ulcer disease (Adj OR 6.8) were risk factors for CDI (Table 3). The Nagelkerke R^2^ for the model was 0.229. Having CDI in the period from July to September inclusive (15 patients) was included in the original model but was not significant.

**Table 3:** Multiple logistic regression analysis for predictors of CDI including only patients with hospital admissions from October 2011 onwards and CDI onset from October 2011.

	Adjusted Odds Ratio	p value	95% CI^1^ for Adjusted Odds Ratio
Neutropenia at any time	15.55	<0.001	7.17, 33.68
Acute lymphocytic leukaemia not in remission	3.22	0.007	1.37, 7.57
Peptic ulcer disease	6.79	0.009	1.62, 28.54
Renal disease	2.83	0.026	1.13, 7.07

^1^ 95% confidence intervals

Model 2 –All patients

ALL not in remission (Adj OR 2.6), neutropenia (Adj OR 12.5), bacterial pneumonia (Adj OR 6.2), other bacterial infections (Adj OR 2.4) and an interaction term between bacterial pneumonia and neutropenia (Adj OR 0.2) were significantly associated with CDI in the model (Table 4). The Nagelkerke R^2^ for the model was 0.230.

**Table 4:** Multiple logistic regression analysis for predictors of CDI with all patients included.

Factor	Adjusted Odds Ratio	p value	95% CI^1^ for adjusted Odds Ratio
Acute lymphocytic leukaemia not in remission^2^	2.60	0.008	1.28, 5.30
Neutropenia at any time	12.50	<0.001	5.47, 28.61
Any bacterial pneumonia	6.23	0.002	1.98, 19.64
Other bacterial infection	2.43	0.004	1.34, 4.42
Interaction term–Any bacterial pneumonia and neutropenia at any time	0.16	0.008	0.04, 0.62

^1^ 95% confidence intervals

^2^ Patients were only classified as being in remission if they were in remission for the full duration of the study.

### Multivariate analysis–Death within 60 and 90 days as the outcome

For both models only patients with CDI or an assigned proxy date (n = 1137) were included. With Cox Proportional Hazards regression, CDI was a risk factor for death within 60 days (Adj HR 35.31). The other risk factors included in the model were AML not in remission, age 65 and over, cerebrovascular disease, dementia and neutropenia (Table 5). CDI correlated with neutropenia and an interaction term between these two variables was also significant.

**Table 5:** Cox proportional hazards regression analysis for predictors of death within 60 days following *C*. *difficile* infection or proxy date.

Factor	Adjusted Hazard Ratio	p value	95% CI^1^ for Adjusted Hazard Ratio	
			Lower	Upper
Acute myeloid leukaemia not in remission^2^	2.91	0.017	1.21	7.03
*C*. *difficile* infection	35.31	<0.001	11.09	112.50
Age 65 and over	4.33	<0.001	1.97	9.53
Cerebrovascular disease	6.78	0.002	1.97	23.31
Dementia	9.23	0.004	2.08	41.05
Neutropenia at any time	2.94	0.015	1.23	7.00
Interaction term—Neutropenia and *C*. *difficile* infection	0.19	0.022	0.04	0.79

^1^ 95% confidence intervals

^2^ Patients were only classified as being in remission if they were in remission for the full duration of the study.

With Cox Proportional Hazards regression, CDI was associated with death within 90 days (Adj HR 22.48). The other risk factors included in the model were ALL not in remission, AML not in remission, age, cerebrovascular disease, dementia, renal disease and neutropenia (Table 6). CDI correlated with neutropenia and an interaction term between these two variables was also significant. The variable renal disease did not meet the proportional hazards assumption and the final model was stratified by renal disease. All other variables met the proportional hazards assumption.

**Table 6:** Cox proportional hazards regression for predictors of death within 90 days following *C*. *difficile* infection or proxy date, stratified by renal disease.

Factor	Adjusted Hazard Ratio	p value	95.0% CI^1^ for Adjusted Hazard Ratio	
			Lower	Upper
Acute myeloid leukaemia not in remission^2^	3.07	0.003	1.47	6.44
*C*. *difficile* infection	20.60	<0.001	8.03	52.89
Age 65 and over	3.08	<0.001	1.70	5.59
Cerebrovascular disease	3.56	0.038	1.07	11.85
Dementia	10.87	<0.001	3.25	36.32
Neutropenia at any time	2.17	0.03	1.09	4.34
Interaction term—Neutropenia and *C*. *difficile* infection	0.27	0.029	0.08	0.87

^1^ 95% confidence intervals

^2^ Patients were only classified as being in remission if they were in remission for the full duration of the study.

## Discussion

Our cohort study of patients in WA with haematological malignancies found that neutropenia was a strong, independent risk factor for CDI in this patient group. CDI was strongly associated with death, as was a history of neutropenia and having either ALL or AML. Thirteen of the 65 CDI cases (20%) either acquired their infection in the community, had an indeterminate source or only had single day admissions within the 28 days prior to infection suggesting that the community is an important source of infection. Our study differed in two ways to other published studies. First, the use of data linkage meant that patients with haematological malignancies who were being managed outside of haematology units not during intensive treatment were included in the study, allowing us to examine the impact of CDI on a greater clinical spectrum of haematological malignancies. Second, the study was undertaken in an area of the world where *C*. *difficile* PCR ribotype 027 has not become established. In spite of such differences, we had similar findings to other studies involving only patients being managed in haematology units.

The incidence of CDI amongst patients with haematological malignancies was 15/10,000 bed days, higher than that reported from studies of general hospitalised patients in Australia at 3.6/10,000 bed days [5]. The increased risk of CDI among patients with haematological malignancies has been recognised for over 25 years [26]. Previous studies have also found a higher incidence of CDI amongst patients with AML [6,7], following autologous and allogeneic stem cell transplants [6,8–11], and amongst patients with neutropenia [25,27]. However the cumulative incidence in our study (3.1%) was lower than in most other studies [7,11,12,23], which may reflect the different patient group in our study through the utilisation of data linkage. For example, Apostolopoulou et al [12] found that the cumulative incidence of CDI amongst patients in a haematology ward over 7 months was 10.8% and Schalk et al [7] found that the cumulative incidence of CDI amongst patients with AML was 18% per patient over a period of 5 years, while another study utilising administrative data to investigate CDI in patients with leukaemia found similar rates to our findings [6]. We found the cumulative incidence of CDI amongst patients undergoing autologous stem cell transplantation to be 11.3% in our study, higher than found in a recent study in the USA [9] and in a meta-analysis that included studies undertaken prior to the emergence of *C*. *difficile* PCR ribotype 027 [28], but lower than in some other studies [29]. This may also reflect the fact that *C difficile* PCR ribotype 027, which has been responsible for an increase in incidence of CDI in hospitals across Northern Europe and North America, has not become established in Australia [30]. Other studies have found that patients with non-Hodgkin’s lymphoma were also at higher risk of CDI compared to other haematological malignancies [12], but we did not find this, perhaps because our study included patients with this malignancy with lower levels of acuity who were being managed outside of haematology units.

We found that neutropenia documented in at least one hospital admission was a strong predictor of CDI. Having a history of a bacterial infection was also significant, which may be a proxy for antibiotics or reflect more severe neutropenia. A number of studies have reported particular risk factors for CDI amongst patients with haematological malignancies [7,8,11,12,23–25]. The use of certain antibiotics, or the intake of a large number of antibiotics [7,11,12,24,25], and certain chemotherapy drugs (particularly those that irritate the gut mucosa) [23,24] were associated with CDI in some studies. Consistent with our findings, neutropenia was associated with CDI amongst patients with haematological malignancies in a number of studies, particularly if for a long duration [7,12,27]. Luo and colleagues [6] found that sepsis was a risk factor for CDI. We also found that having renal disease and peptic ulcer disease, but not diabetes, were independent risk factors for CDI. In their study of risk factors for community-acquired CDI, Dial and colleagues also found renal disease to be a risk factor, as well as the use of proton-pump inhibitors, but not peptic ulcer disease [31]. To our knowledge, the association between peptic ulcer disease and CDI in patients with a haematological malignancy has not been previously described. Unfortunately, we were not able to examine whether antimicrobials, chemotherapy drugs or proton-pump inhibitors were associated with CDI in our study population as these data are not collected electronically in the HMDS.

We speculate that the duration and severity of neutropenia may be important predictors of CDI in this patient group. Our study did not enable quantification of neutropenia or its duration. Curative treatment for AML involves cycles of chemotherapy resulting in neutropenia for 3 weeks out of 4 and, during palliation, patients with AML often experience chronic neutropenia [32,33]. Therefore our finding that almost 61% of AML patients had neutropenia documented in at least one hospital admission is not surprising, and perhaps is an underestimate. Neutropenia of short duration may not be documented in the hospital records and it is possible that documentation of this condition is incomplete in our data.

In our study, CDI was consistently associated with increased risk of death, as documented elsewhere [6,34,35]. However, we also found that CDI was rarely documented as a reason for death. In addition, 25 of the 65 CDI cases did not have CDI listed as a reason for hospital admission, and therefore CDI was not documented as having contributed to the patients’ duration of hospital stay. Neutropenia along with CDI was associated with increased risk of death. Yoon and colleagues also found that neutropenia was an independent predictor of mortality amongst patients with both CDI and malignancy. In addition, they found that the majority of patients who died (49/61 patients) did not have severe CDI [36]. We therefore postulate that, while CDI directly contributes to death in some patients with haematological malignancies, in others the increased risk of death amongst patients with CDI may be due to some factors that predispose to both CDI and death, rather than through direct causation.

The vast majority of our CDI cases (62) were classified as hospital acquired infections. However, of these, 10 only had single day admissions in the 28 days prior to onset. This raises the question as to whether or not these 10 cases actually acquired their infection in hospital or whether their source was in the community. In contrast to patients without haematological malignancies, patients with haematological malignancies who acquire CDI appear to be infected with a wider variety of *C*. *difficile* strains and these strains tend to be those that are observed to be circulating in the community [34]. One hypothesis to explain this observation is that patients with haematological malignancies are more likely to acquire CDI from sources in the community by virtue of their impaired immunity. Although ribotyping data were only available on approximately half our cases, the diversity of RTs found suggests that there are many sources of infection. A study of CDI amongst patients undergoing allogeneic stem cell transplantation also found a diversity of *C*. *difficile* strains apart from the dominant strain *C difficile* PCR ribotype 027 [37]. Thus, community-acquired CDI may be under-estimated in this patient group.

The major limitation of our study was that we did not have access to clinical details such as drugs administered, specific laboratory data on each patient, and the duration of the primary disease. We also only had access to 1 year of data, and the total number of CDI cases was small compared to some other studies.

In spite of the limitations to our study, our findings were consistent with the findings of other studies using different data sources. Our study does provide a useful insight into the risk factors for and impact of CDI amongst Australian patients with haematological malignancies, and raises the possibility that community acquisition of CDI may be under-estimated

## Conclusions

Neutropenia was strongly associated with CDI. While having CDI is a risk factor for death, in many cases it may not be a direct contributor to death but may reflect patients having greater morbidity. More work is needed to best understand the pathogenesis of CDI in this group of patients, and the mode of contact and spread, in order to reduce the burden of disease and mortality in this vulnerable patient group.

## Supporting Information

S1 Table Characteristics of the study sample by CDI status–potential predictors, all Charlson’s comorbidities. (DOCX)

S2 Table *Clostridium difficile* ribotypes. (DOCX)
