# Supplementary material for: Clostridium difficile Infections amongst Patients with Haematological Malignancies: A Data Linkage Study
Source: PLoS One. 2016 Jun 17;11(6):e0157839. doi: 10.1371/journal.pone.0157839 (PMC4912117; doi:10.1371/journal.pone.0157839)
Supplement: S1 Table — (DOCX) [file pone.0157839.s001.docx]

**S1 Table. Characteristics of the study sample by CDI status – potential predictors – all Charlson’s comorbidities**

| **Type of Charlson’s comorbidity** |  | **CDI^1^**  **n (%)** | **p value^2^** |
| --- | --- | --- | --- |
|  | Total number of patients | **65 (3.1)** |  |
| Acute myocardial infarction | 31 | 3 (9.7) | .07 |
| Congestive heart failure | 119 | 4 (3.4) | 0.750 |
| Peripheral vascular disease | 21 | 0 | 1.0 |
| Cerebrovascular disease | 34 | 1 (2.9) | 1.0 |
| Dementia | 20 | 0 | 1.0 |
| Chronic obstructive pulmonary disease | 92 | 3 (3.3) | 0.763 |
| Rheumatoid disease | 7 | 0 | 1.0 |
| Peptic ulcer disease | 21 | 3 (14.3) | 0.026 |
| Mild liver disease | 24 | 0 | 1.0 |
| Diabetes | 55 | 1 (1.8) | 1.0 |
| Diabetes with complications | 89 | 5 (5.6) | 0.197 |
| Hemiplegia or paraplegia | 20 | 2 (10.0) | 0.127 |
| Renal disease | 121 | 8 (6.6) | 0.051 |
| Moderate/severe liver disease | 21 | 2 (9.5) | 0.138 |
| Metastatic cancer | 45 | 1 (2.2) | 1.0 |
| AIDS | 5 | 0 | 1.0 |

^1^ Patients who had *C. difficile* infection at least once in the study period

^2^ Outcome of the test for significance of difference between patients with and without *C. difficile* infection using a Chi squared test or Fisher’s exact test for categorical variables
