# Supplementary material for: Clostridium difficile Infections amongst Patients with Haematological Malignancies: A Data Linkage Study
Source: PLoS One. 2016 Jun 17;11(6):e0157839. doi: 10.1371/journal.pone.0157839 (PMC4912117; doi:10.1371/journal.pone.0157839)
Supplement: S2 Table — (DOCX) [file pone.0157839.s002.docx]

**S2 table. *Clostridium difficile* ribotypes**

| Ribotype | | Number of cases |
| --- | --- | --- |
|  | UK 014/020 [G] | 7 |
|  | UK 002 | 5 |
|  | UK 017 | 3 |
|  | UK 056 | 2 |
|  | UK 244 | 2 |
|  | QX 033 | 1 |
|  | QX 077 | 1 |
|  | QX 097 | 1 |
|  | QX 135 | 1 |
|  | QX 142 | 1 |
|  | QX 150 | 1 |
|  | UK 003 | 1 |
|  | UK 005 | 1 |
|  | UK 015 / UK 193 | 1 |
|  | UK 018 | 1 |
|  | UK 051 | 1 |
|  | UK 054 | 1 |
|  | UK 070 | 1 |
|  | UK 103 | 1 |
|  |  |  |
|  | **Total** | **33** |
